# Chronic Kidney Disease Interplay with Comorbidities and Carbohydrate Metabolism: A Review

**DOI:** 10.3390/life14010013

**Published:** 2023-12-21

**Authors:** Radha Kushwaha, Pothabathula Seshu Vardhan, Prem Prakash Kushwaha

**Affiliations:** 1 Centre of Food Technology, University of Allahabad, Allahabad 211002, Uttar Pradesh, India; radhakushwahagunn@gmail.com; 2 Department of Chemistry, Sardar Vallabhbhai National Institute of Technology (SVNIT), Surat 395007, Gujarat, India; seshu.target@gmail.com; 3 Department of Biological, Geological, and Environmental Sciences, Cleveland State University, Cleveland, OH 44115, USA

**Keywords:** chronic kidney disease, cellular growth homeostasis, inflammation, insulin resistance, dietary interventions

## Abstract

Chronic kidney disease (CKD) poses a global health challenge, engendering various physiological and metabolic shifts that significantly impact health and escalate the susceptibility to severe illnesses. This comprehensive review delves into the intricate complexities of CKD, scrutinizing its influence on cellular growth homeostasis, hormonal equilibrium, wasting, malnutrition, and its interconnectedness with inflammation, oxidative stress, and cardiovascular diseases. Exploring the genetic, birth-related, and comorbidity factors associated with CKD, alongside considerations of metabolic disturbances, anemia, and malnutrition, the review elucidates how CKD orchestrates cellular growth control. A pivotal focus lies on the nexus between CKD and insulin resistance, where debates persist regarding its chronological relationship with impaired kidney function. The prevalence of insulin abnormalities in CKD is emphasized, contributing to glucose intolerance and raising questions about its role as a precursor or consequence. Moreover, the review sheds light on disruptions in the growth hormone and insulin-like growth factor axis in CKD, underscoring the heightened vulnerability to illness and mortality in cases of severe growth retardation. Wasting, a prevalent concern affecting up to 75% of end-stage renal disease (ESRD) patients, is analyzed, elucidating the manifestations of cachexia and its impact on appetite, energy expenditure, and protein reserves. Taste disturbances in CKD, affecting sour, umami, and salty tastes, are explored for their implications on food palatability and nutritional status. Independent of age and gender, these taste alterations have the potential to sway dietary choices, further complicating the management of CKD. The intricate interplay between CKD, inflammation, oxidative stress, and cardiovascular diseases is unraveled, emphasizing the profound repercussions on overall health. Additionally, the review extends its analysis to CKD’s broader impact on cognitive function, emotional well-being, taste perception, and endothelial dysfunction. Concluding with an emphasis on dietary interventions as crucial components in CKD management, this comprehensive review navigates the multifaceted dimensions of CKD, providing a nuanced understanding essential for developing targeted therapeutic strategies.

## 1. Introduction

CKD is a growing concern that can result in kidney failure and ESRD. It is impacting populations worldwide causing health complications and increasing the risk of serious illnesses, with high mortality rates [1,2,3,4,5,6]. CKD is associated with different complex deleterious changes in a patient’s physiology and metabolic activity. They include deteriorating function and/or subsequent kidney failure, uremia, irregularities in metabolism of amino acid, lipids, minerals, and homocysteine (leads to malnutrition, anemia, vitamin deficiency, dementia, stroke and heart diseases), metabolic acidosis, insulin resistance, inflammatory and oxidative stress, dysfunction of skeletal muscle and many more [7,8,9,10,11]. Further, other diseases or disease-causing factors (diabetes and hypertension) which coexist within CKD are associated with deteriorating the health and mortality [7].

Carbohydrate metabolism in the human body involves glycolysis, the tricarboxylic acid (TCA) cycle, and insulin-regulated processes for energy balance [12]. The liver regulates glucose levels through glycogenesis, glycogenolysis, and gluconeogenesis. Kidneys play a role in glucose homeostasis via sodium glucose co-transporters (SGLT)-mediated reabsorption. CKD and metabolic issues have a bidirectional impact, causing insulin resistance and cardiovascular complications. CKD-induced insulin resistance leads to skeletal muscle glucose uptake reduction and increased liver gluconeogenesis [13].

Insulin, a hormone secreted by pancreatic β-cells, plays a pivotal role in regulating glucose homeostasis, energy balance, and various aspects of lipid, protein, and mineral metabolism. In cases of insulin resistance, a clinical condition marked by reduced biological responsiveness to insulin at any given blood concentration, there is a noteworthy connection to CKD according to various epidemiologic studies [14]. Debates persist regarding whether insulin resistance serves as a precursor to CKD or arises as a consequence of impaired kidney function. Nevertheless, it is noteworthy that insulin resistance prevails in individuals with ESRD and those with moderate-to-advanced CKD. Additionally, irregularities in insulin secretion can lead to glucose intolerance in such cases. The underlying causes of these insulin abnormalities may encompass factors such as uremic toxins stemming from protein catabolism, vitamin D deficiency, metabolic acidosis, anemia, inadequate physical fitness, and cachexia [15].

Furthermore, it is important to note that CKD can cause disruptions in the growth hormone (GH) and insulin-like growth factor (IGF 1) axis. This disruption often leads to growth in children. It is worth mentioning that when CKD is accompanied by, to severe growth retardation there is an increased risk of illness and mortality [16].

Wasting is a prominent issue observed in individuals with CKD. Research indicates that as many as 75% of adults with ESRD undergoing maintenance dialysis exhibit signs of wasting [17]. The wasting syndrome, also known as cachexia, in CKD patients is characterized by symptoms such as reduced appetite, heightened energy expenditure, diminished protein reserves evidenced by low serum albumin levels, and both weight and muscle mass loss [18].

Patients with CKD experience endocrine system changes influenced by multiple factors. The kidney’s dual role in hormone breakdown and synthesis contributes to this phenomenon. Coexisting conditions such as inflammation, metabolic acidosis, and malnutrition also play a role in the development of endocrine abnormalities in CKD patients [18]. Notable deficiencies include calcitriol, testosterone, insulin-like growth factor, and erythropoietin, while hormones like prolactin, growth hormone, and insulin often accumulate. These endocrine alterations lead to clinical consequences such as anemia, infertility, and bone diseases. Advancement to later stages of CKD has been linked to a notable rise in the production of free radicals and prooxidants. Multiple studies have demonstrated elevated plasma markers of oxidative stress in CKD patients, signifying heightened systemic oxidative stress [19,20,21].

Various researchers have reported that CKD is associated with disruptions in functionality, such as cognitive dysfunction, endothelial dysfunction and also involved in emotional and taste perceptions. From this perspective, we reviewed the potential effect of CKD on human health and diet therapy that may reduce the severity of the CKD. We have searched the PubMed, Google Scholar and Scopus database to search the relevant studies associated with CKD, CKD-linked comorbidities and carbohydrate metabolism.

## 2. Carbohydrate Metabolism and Its Impairment in CKD Patients

Carbohydrate metabolism is a fundamental process that regulates the utilization of glucose, a primary energy source, in the human body. This intricate system involves various biochemical pathways and is tightly regulated to maintain energy homeostasis [22]. The process begins with glycolysis, occurring in the cytoplasm, where glucose is broken down into pyruvate, generating a small amount of adenosine triphosphate (ATP) and nicotinamide adenine dinucleotide (NADH). Pyruvate can then enter the mitochondria for further metabolism in the TCA cycle, contributing to the production of more ATP through oxidative phosphorylation [23]. Insulin, a hormone secreted by the pancreas, plays a crucial role in glucose metabolism. Upon elevated blood glucose levels, insulin facilitates glucose uptake by cells, promoting its conversion to glycogen for storage in the liver and muscles. This process is known as glycogenesis, serving as a short-term energy reserve. Conversely, during fasting or periods of low glucose, glycogen undergoes glycogenolysis, releasing glucose back into the bloodstream to maintain blood glucose levels. The liver, in particular, plays a key role in regulating systemic glucose levels by balancing glycogen storage and release. In instances where glucose is scarce, gluconeogenesis occurs, predominantly in the liver and kidneys. This process synthesizes glucose from non-carbohydrate precursors, such as amino acids and glycerol, ensuring a steady supply of glucose even in fasting states [24].

The kidneys play a crucial role in glucose homeostasis by filtering and reabsorbing glucose from the glomerular filtrate. Proximal tubular cells, particularly in the S1 and S2 segments, actively reabsorb glucose through SGLT. This cotransport mechanism couples the uphill movement of sodium ions with the reabsorption of glucose. Subsequently, glucose exits the tubular cells via facilitative glucose transporters (GLUTs), returning to the bloodstream. The SGLT-mediated reabsorption ensures the efficient retrieval of glucose, preventing its excretion and maintaining glucose homeostasis in the body [25]. CKD and metabolic disturbances share a bidirectional relationship. CKD induces insulin resistance, dyslipidemia, and inflammation, promoting diabetes and cardiovascular complications. Simultaneously, metabolic abnormalities exacerbate kidney dysfunction. This intricate interplay emphasizes the importance of holistic management strategies addressing both CKD and metabolic disorders to improve patient outcomes [26]. Insulin resistance, a hallmark of CKD, arises when cells exhibit diminished responsiveness to insulin’s regulatory signals. CKD-induced factors, including inflammation, oxidative stress, and altered hormonal regulation, contribute to insulin resistance. Skeletal muscle, a major insulin target, experiences reduced glucose uptake, while the liver increases gluconeogenesis, elevating blood glucose levels. Insulin resistance in CKD is linked to metabolic complications, such as impaired glucose tolerance and an increased risk of developing diabetes mellitus. Managing insulin resistance becomes crucial in mitigating the progression of both CKD and associated metabolic disorders in affected individuals [27].

In a study, incidence of insulin resistance (IR) and assess impaired fasting glucose (IFG), impaired glucose tolerance (IGT), and diabetes mellitus (DM) in CKD patients was studied. Among 113 CKD patients, 59.3% had IFG, and 69% exhibited a carbohydrate metabolism disorder. Additionally, 23% of CKD patients had IR, with elevated levels of fasting blood glucose, insulin, triglycerides, and HbA1c. Notably, 31.3% of patients with IFG were found to have IR. The findings highlight the prevalence of metabolic disorders and insulin resistance in CKD patients, emphasizing the importance of screening for diabetes in this population [28].

## 3. CKD Controls Cellular Growth Homeostasis

Children suffering from chronic renal failure (CRF) often experience stunted growth, and disruptions in the GH/IGF axis may contribute to this growth impairment. In the context of CKD in children, there are notable changes in the GH and IGF-1 axis, which lead to growth retardation. Importantly, renal failure in these cases is characterized by GH resistance rather than GH deficiency [16].

Information derived from previous studies conducted by the North American Pediatric Trials and Collaborative Studies suggests that around 37% of pediatric CKD patients encounter legal growth limitations [29,30]. Notably, there is a significant correlation between growth impairment and the age at which patients are enrolled in the studies. Infants (aged 0–2 years) and young children exhibit average height standard deviation scores (HtSDS) of −2.33 and −1.65, respectively, whereas adolescents have an HtSDS of only −0.93 [31].

After infancy and early childhood, the main cause of growth deficiency is typically associated with disturbances in the metabolism of GH and its primary mediator, IGF-I [32,33]. GH resistance stems from various mechanisms, including compromised Janus kinase/signal transducer and activator of transcription (JAK/STAT) signaling following GH receptor activation, lower GH receptor density in target organs, and a decrease in free IGF-1 levels due to elevated levels of inhibitory IGF-binding proteins (IGFBPs).

- It is worth noting that the GH/IGF system is partially influenced by calorie and protein intake, and inadequate nutrition can therefore result in compromised growth [34]. In CKD, a wasting or cachectic syndrome is observed, and while the mechanisms behind cachexia are intricate, they encompass factors such as anorexia, nausea, vomiting, increased basal metabolic rate, loss of lean body mass, and reductions in serum proteins like albumin, transferrin, and prealbumin [17]. Other factor such as genetic factors also play a role in determining a child’s height potential even if they may inherit height traits from their parents [35]. Gender can influence growth patterns, with boys typically experiencing growth spurts during adolescence, while girls tend to grow earlier and at a steadier pace [36]. Certain genetic syndromes like syndromic kidney diseases affecting the kidneys can have a direct impact on growth due to the underlying genetic mutations [37]. Birth-related factors including premature birth may lead to delayed development and lower birth weights in infants [38]. Those born small for their gestational age could face growth restrictions due to intrauterine factors. Infants requiring intensive care after birth may experience growth challenges from medical interventions and associated stress. Prematurity can result in delayed development and lower birth weights, impacting infant growth [38]. Growth restrictions may affect infants born small for gestational age due to intrauterine factors, influencing their overall development. Other comorbid health conditions involving the central nervous system, liver, or heart can impact a child’s overall health and growth [39]. The age at which CKD begins can affect growth patterns, as children who develop CKD at a younger age may experience more significant growth issues [40]. The extent of kidney damage and the level of residual renal function can influence growth in children undergoing dialysis treatments. Metabolic disturbances such as salt and water metabolism imbalance can lead to fluid retention or dehydration that affect growth [41]. Metabolic acidosis, characterized by acidic blood pH, can affect both bone health and growth. In CKD-Mineral and Bone Disorder (MBD), disruptions in mineral and bone metabolism may contribute to issues with growth [42]. Anemia, a common complication of CKD, can lead to fatigue and reduced physical activity, potentially affecting growth [43]. Various factors can contribute to malnutrition in CKD, including altered taste sensation, anorexia (loss of appetite), vomiting, dietary restrictions, and nutrient losses during dialysis [44]. Protein-energy wasting is a complex issue involving factors such as infections, inflammation, uremic toxins, oxidative stress, and inflammatory cytokines [45]. These can collectively impair growth and nutritional status.

## 4. Hormonal Disturbances

When a child’s kidney function, specifically the glomerular filtration rate (GFR), starts to decline, it has a significant impact on their growth. This impact becomes especially noticeable when the GFR falls below 90 mL/min/1.73 m^2^, and becomes even more severe when it drops below 25 mL/min/1.73 m^2^. For children with early onset CRF, both their prenatal and postnatal growth can be affected, leading to significant growth issues even before they reach the age of 3, especially when compared to older children. Among different age groups, younger children tend to experience more profound growth challenges due to CRF. Furthermore, children in CKD stages 2–4 develop metabolic issues that further hinder their growth. The extent of their kidney disease is directly linked to the degree of growth failure, with patients in the ESRD category experiencing the most significant height limitations [46,47,48]. As the GFR falls below 60 mL/min, additional metabolic complications, such as anemia, secondary hyperparathyroidism, metabolic acidosis, renal bone disease, and increased sodium excretion, worsen. These complications further compromise the child’s ability to achieve normal growth and development [29,49].

Hence, the primary factor that seems to contribute to growth problems in children with CKD, particularly at a young age, is inadequate nutrition, often due to issues such as loss of appetite or vomiting. Studies have demonstrated that maximizing calorie intake to at least 80% of their nutritional requirements can effectively enhance growth in children who develop CKD during infancy [50,51,52,53]. According to a widely agreed-upon document discussing the use of recombinant human growth hormone (rhGH) in CKD, it is recommended to consider rhGH treatment for all children whose height falls below the third percentile or whose height growth rate is below -2 standard deviations (SD), after accounting for metabolic and nutritional issues [54]. Research findings indicate that using rhGH after a kidney transplant can help children experience catch-up growth. This growth is linked to an increase in their IGF-1 levels [55].

In adults, several hormones, and their impaired function in CKD lead to hormonal imbalances with wide-ranging consequences for adults [56]. The Renin-Angiotensin-Aldosterone System (RAAS) is a key player in blood pressure regulation. In CKD, the kidneys’ diminished ability to filter blood triggers the release of renin [57]. This initiates a cascade resulting in the production of angiotensin II and aldosterone. Angiotensin II causes blood vessel constriction, and aldosterone leads to sodium and water retention, contributing to hypertension. Chronic elevation of blood pressure can exacerbate kidney damage, forming a detrimental cycle that further impairs kidney function [58]. The kidneys produce erythropoietin (EPO), a hormone that stimulates the production of red blood cells in the bone marrow. In CKD, declining kidney function results in reduced EPO production, leading to anemia [59]. Anemia in CKD is associated with fatigue, weakness, and decreased oxygen-carrying capacity. Treatment often involves erythropoiesis-stimulating agents (ESAs) to stimulate red blood cell production. Addressing anemia is crucial not only for improving quality of life but also for reducing the cardiovascular strain associated with CKD [60]. The kidneys play a pivotal role in converting vitamin D into its active form, calcitriol. In CKD, impaired kidney function leads to reduced production of calcitriol [61]. This deficiency disrupts calcium and phosphorus metabolism, contributing to bone disorders such as renal osteodystrophy. The decline in calcitriol also triggers secondary hyperparathyroidism, wherein the parathyroid glands release more parathyroid hormone (PTH) to maintain calcium balance. Elevated PTH levels contribute to bone resorption, further impacting bone health in CKD patients [62]. CKD often results in secondary hyperparathyroidism due to elevated PTH levels. The increased secretion of PTH is a compensatory mechanism to regulate calcium and phosphorus levels. However, persistent elevation can lead to bone disease, causing bone pain, fractures, and deformities. Managing secondary hyperparathyroidism involves addressing mineral and bone disorders through dietary interventions, phosphate binders, and medications to regulate PTH levels [62]. CKD can impact reproductive hormones, affecting both men and women. In men, CKD may lead to reduced testosterone levels, contributing to symptoms such as fatigue, decreased muscle mass, and sexual dysfunction. In women, irregular menstrual cycles and hormonal imbalances can affect fertility. The hormonal disruptions in CKD may result from a combination of factors, including inflammation, oxidative stress, and alterations in hormone clearance by the impaired kidneys [63]. Management may involve hormone replacement therapy and addressing underlying factors contributing to hormonal imbalances [64]. CKD can also influence thyroid function. Thyroid hormones play a crucial role in metabolism, and alterations in thyroid function can impact energy levels and body weight [65]. Hypothyroidism, characterized by low thyroid hormone levels, can occur in CKD and may contribute to symptoms such as fatigue, weight gain, and cold intolerance. Managing thyroid function is essential to maintaining overall metabolic balance [65].

In summary, although rhGH therapy is often needed for many children with CKD, ensuring they grow properly requires a holistic approach that takes into account both their nutrition and metabolic health in managing the condition effectively. In adults, hormonal imbalances result in widespread consequences including hypertension, anemia exacerbating kidney damage. Impaired kidney function in CKD causes deficiencies in calcitriol, triggering secondary hyperparathyroidism and impacting bone health, necessitating management strategies.

## 5. Role of CKD on Wasting and Malnutrition

CKD, characterized by the progressive decline in renal function, is an escalating health concern with potential public health implications. Malnutrition and wasting are prevalent issues in this patient population, significantly impacting morbidity, mortality, functional abilities, and overall quality of life [66,67].

Malnutrition typically refers to a poor nutritional state resulting from inadequate nutrient intake. However, in the context of CKD, major malnutrition can also stem from insufficient food intake, coupled with low levels of serum and tissue proteins, irrespective of body weight adherence to standard nutritional guidelines [68]. Various studies in children with CKD have reported a prevalence of malnutrition ranging from 20% to 45%, depending on the clinical criteria used [69,70,71]. Using the subjective global assessment scale (SGA), a recent study found that 31% of adults with CKD, including both dialysis and non-dialysis patients, experienced protein-energy wasting [72].

**Figure 1:**
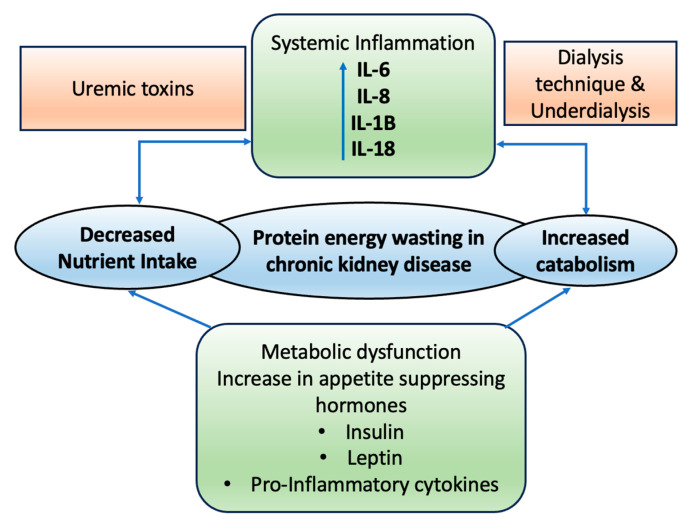
Illustration depicting the factors leading to protein-energy wasting and the pathophysiological interactions in chronic kidney disease. IL = Interleukins.

Wasting syndromes exhibit maladaptive reactions, such as loss of appetite and increased metabolism, which can lead to low serum albumin levels, weight loss, decreased muscle mass, and, intriguingly, the preservation or even an increase in fat mass (Figure 1). This complexity arises from various contributing factors [17,73]. The Society for Cachexia and Wasting Disorders (SCWD) describes cachexia or wasting as a complicated metabolic condition associated with underlying illnesses. It is marked by the loss of muscle, sometimes alongside the loss of fat [74]. In individuals with CKD, wasting can occur due to several reasons, such as not eating enough nutritious food, inflammation throughout the body, disruptions in hormonal balance, and irregular signaling from neuropeptides in the body [17,73,75,76,77,78,79,80].

The likelihood of muscle loss in CKD patients is linked to the stage of their condition. As CKD advances to stages 4 and 5, a decline in nutritional intake often goes hand in hand with worsening protein and energy levels in the body. This means that as kidney disease becomes more severe, people may struggle more with their nutrition and energy levels, which can contribute to muscle wasting [81]. As a result, muscle loss becomes more common in the later stages of kidney disease. In adults, a significant degree of muscle wasting (in stages 3 and 4) can be found in a range of 18% to 48%, and it can even climb as high as 75% in individuals with end-stage renal disease. This means that as kidney disease progresses, more and more people are affected by muscle loss, and the numbers can be quite substantial in severe cases [81,82,83].

**Figure 2:**
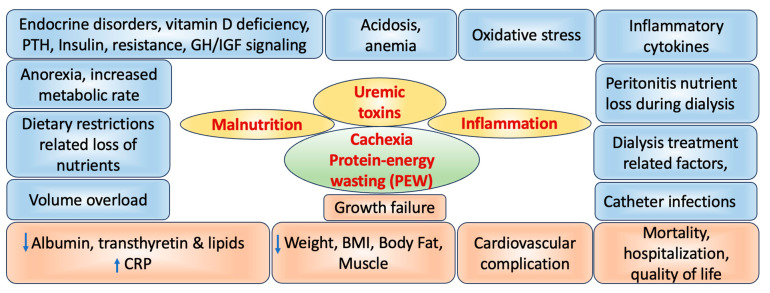
Schematic representation of protein-energy wasting (PEW) and the pathophysiological effects associated with kidney disease. BMI = Body mass index; CRP = C-reactive protein. Upward arrow = Increased levels; Downward arrow = Reduced levels.

The pathogenesis of malnutrition and wasting in patients with CKD is multifactorial, with several risk factors contributing to these conditions. Figure 2 representing the overview of the various factors involved in the pathophysiology of the CKD.

Muscle protein degradation, which is responsible for muscle protein wasting, involves four major proteolytic pathways. These pathways include the ATP-dependent ubiquitin–proteasome system and the cathepsin system, which can facilitate comprehensive breakdown of cellular proteins into amino acids or small peptides. In contrast, the caspase-3 and cytosolic calcium-dependent calpain systems have limited proteolytic capabilities due to their restricted specificity [84].

## 6. Association of CKD Inflammation, Oxidative Stress and Cardiovascular Diseases

Inflammation and oxidative stress are common in patients with CKD and can exacerbate their clinical symptoms. Oxidative stress occurs when there is a disruption in the balance between harmful substances like reactive oxygen species (ROS) or reactive nitrogen species (RNS) and protective antioxidants in the body. These harmful substances, because of their molecular instability (such as having unpaired electrons), tend to trigger oxidation reactions with different molecules like proteins, lipids, and DNA in an attempt to stabilize themselves [85].

Key ROS include free radicals like hydroperoxide, the highly reactive hydroxyl radical and superoxide anion radical. Additionally, there are redox signaling agents like hydrogen peroxide (H_2_O_2_) and singlet oxygen (^1^O_3_), which, although lacking unpaired electrons, possess significant oxidizing properties. Oxidative stress reactions can also include oxygen compounds that team up with other elements, not just free radicals like the alkoxy radical, but also non-radical compounds such as peroxynitrite (ONOO^−^) and hypochlorous acid (HOCl). These reactions demonstrate how various molecules can become involved in oxidative stress, not just those with unpaired electrons [86].

Oxidative stress can manifest through four distinct pathways: chlorinated stress, carbonyl stress classical oxidative stress, and nitrosative stress [87]. People with CKD/ESRD often experience higher levels of oxidative stress because their natural defense mechanisms against it are not working as well as they should. This heightened oxidative stress can lead to nucleic acid damage, increasing the risk of developing subsequent tumors. Oxidative stress in kidney and vascular tissues can also contribute to hypertension, and vice versa, creating a vicious circle of oxidative stress and hypertension [88].

Several factors contribute to heightened oxidative stress in individuals with CKD, including gut dysbiosis, hyperglycemia, dialysis, and inflammation [89,90,91,92].

CKD is known to be a condition marked by widespread inflammation, which is closely linked to increased oxidative stress. When the body experiences inflammation, cells that fight off infections release harmful reactive substances at these inflamed sites. On the flip side, the byproducts of oxidation and ROS encourage immune cells like macrophages and neutrophils to release inflammatory molecules and even more ROS. This sets up a continuous cycle where inflammation and oxidative stress fuel each other, creating a feedback loop. When immune cells, known as phagocytic cells, release ROS, they can prompt nearby non-immune cells to release inflammatory substances [93]. This means that when something causes a rise in oxidative stress, it can also trigger an inflammatory reaction, which in turn worsens oxidative stress, creating a back-and-forth cycle. Inflammation is characterized by increased levels of markers of inflammation, such as cytokines, acute phase proteins, and adhesion molecules. In this process, cells responsible for the body’s initial immune response play a central role. Many factors play a part in the ongoing state of inflammation in CKD, including things like the body making more inflammatory substances, oxidative stress, acidity in the body, frequent and long-lasting infections, disruptions in gut bacteria, and changes in how fat tissue works [94].

Research involving patients has shown connections between markers of inflammation and various complications associated with CKD. These complications include problems like malnutrition, calcium buildup in the coronary arteries, hardening of the arteries, irregular heart rhythms (atrial fibrillation), an enlarged left ventricle in the heart (left ventricular hypertrophy), heart failure, and a higher risk of death in CKD patients [95,96,97]. Furthermore, inflammation plays a role in worsening CKD, making the body less responsive to insulin, impairing the function of blood vessel linings, affecting the balance of minerals and bones, causing anemia, and making the body less receptive to erythropoietin (a hormone involved in red blood cell production) [98,99,100,101]. 

Chronic inflammation triggers oxidative stress, which is linked to an intensified oxygen reaction when immune cells like monocytes/macrophages and neutrophils are activated. The respiratory burst is a vital weapon in the body’s defense against harmful invaders like bacteria, fungi, and parasites. However, when the body is stuck in a state of ongoing inflammation, immune cells are overstimulated and become overzealous, producing an excessive amount of ROS. In CKD, the proinflammatory factors present cause oxidative stress levels to rise, and the body’s disrupted balance of redox compounds amplifies inflammation even more. These two processes effectively feed off each other, making the situation worse. For example, when the body releases pro-inflammatory cytokines like interleukin 6 (IL-6), it triggers an increase in the expression of Nox4. Conversely, elevated Nox4 expression stimulates the synthesis of IL-6 [102].

The connection between oxidative stress and inflammation is intricate and can work through different pathways, extending beyond well-known proinflammatory substances like tumor necrosis factor-alpha (TNF-α) or IL-6. For example, in the group undergoing hemodialysis (HD), researchers like Uchimura et al. (2001) and Santhanam et al. (2014) discovered a notable connection between advanced glycation end products (AGEs), specifically substances like pentosidine and N(6)-carboxymethyl lysine (which result from both the breakdown of fats and glycation reactions), and the levels of two proinflammatory cytokines: interleukin 18 (IL-18) and macrophage colony-stimulating factor (M-CSF) [103,104].

Several mechanisms are known to underlie the connection between elevated oxidative stress and the progression of both CKD and cardiovascular disease (CVD) in CKD patients. When a patient has kidney failure, it becomes an especially risky situation, particularly if they already had cardiovascular disease. Even in individuals with high blood pressure without kidney problems, there is a connection between their blood creatinine levels and the risk of heart and blood vessel issues, even when those creatinine levels are considered normal [85].

In a study with 1829 patients who had high blood pressure and healthy kidney function, the researchers found that over an 11-year period of monitoring, a rise in blood creatinine levels by 0.23 mg/dL was linked to a 30% increased likelihood of experiencing cardiovascular problems like heart attacks and strokes [105].

## 7. Effect of CKD on Functioning

### 7.1. Cognitive Dysfunction

Individuals dealing with CKD and ESRD are much more likely to experience cognitive difficulties compared to the general population, even when considering factors like age, diabetes, cardiovascular health, and other underlying health conditions as noted by Hailpern et al. (2007) and Yaffe et al. (2010) [106,107]. Additionally, multiple studies have established a link between dementia, mild cognitive impairment, and higher mortality rates among ESRD patients. For instance, in a study conducted by Kurella and colleagues in 2006 [108], they found that among patients undergoing HD, those who had dementia had a higher risk of death. Specifically, they reported that the risk of mortality was 1.48 times higher (with a confidence interval of 95%, ranging from 1.32 to 1.66) for HD patients with dementia compared to those without dementia. Similarly, in a study led by Griva and her team in 2010 [109], they followed 145 patients receiving dialysis who had not previously experienced dementia or strokes. Their findings showed that when comparing patients with mild-to-moderate cognitive issues to those without any cognitive deficits, there was an adjusted hazard ratio for all-cause mortality of 2.53. This means that the risk of death over 7 years was 2.53 times higher (with a 95% confidence interval ranging from 1.03 to 6.22) for those with cognitive impairment compared to those without such deficits.

It is important to note that routine cognitive testing is not typically conducted among CKD and ESRD patients. As a result, the prevalence of cognitive impairment in this population is estimated to range from 30% to 70% and is closely linked to the severity of kidney disease [110,111]. In a study led by Murray and colleagues in 2006 [112], they examined 338 hemodialysis patients aged 55 years or older. Surprisingly, only 2.9% of them had a history of prior cognitive impairment. However, when they assessed these patients for memory, executive function, and language skills, they found that 13.1% had mild cognitive issues, 36.1% had moderate impairment, and 37.3% had severe impairment. Shockingly, only 12.7% of the participants showed normal cognitive function. The exact pathophysiology of dementia and cognitive impairment in CKD remains incompletely understood but is likely multifactorial.

Apart from factors like toxins and metabolic imbalances that can lead to cognitive problems, it is important to consider the role of cerebrovascular disease, which can be a significant contributor. Cerebrovascular disease is more common in people with all stages of CKD compared to those without kidney problems, especially in individuals who are on long-term dialysis treatments. Although some of the higher risk of stroke in CKD and ESRD patients can be linked to common risk factors like diabetes, high blood pressure, cardiovascular disease, and prior strokes, it is important to note that these typical risk factors do not entirely explain the significantly increased risk observed in these individuals [113,114].

Apart from strokes, when researchers conducted brain magnetic resonance imaging (MRI) on dialysis patients, they found signs of cerebral atrophy and other issues in the white matter of the brain. These white matter problems have been linked to cognitive difficulties, even in patients who have not had a stroke before [115]. Individuals with ESRD appear to undergo changes in the structure of their blood vessels and experience increased stiffness in their arteries. These changes may be connected to factors such as having too much fluid in their bodies and disruptions in how calcium and phosphate are managed. Furthermore, the fluctuations in blood flow that occur during HD sessions can potentially make matters worse, possibly causing problems with blood supply to the brain and increasing the risk of stroke or other brain injuries [116,117].

In a cross-sectional study comparing 45 maintenance dialysis patients to 67 controls without known kidney disease, patients undergoing hemodialysis showed higher rates of white matter problems, brain shrinkage, and a significant presence of unnoticed areas of damage in the brain when compared to individuals in the control group [115]. Additionally, in research by Chou et al. (2013) [118], using advanced magnetic resonance imaging (MRI) methods that can detect subtle changes, researchers found that individuals with ESRD had more damage to the protective covering of nerve fibers (axonal demyelination) compared to people of a similar age who were healthy.

### 7.2. On Emotional Functioning

People dealing with CKD can experience an increased risk of developing or worsening psychological issues like depression and anxiety. This often happens because they face various stressors that take a toll on their emotional well-being. These stressors can involve adapting to strict dietary and fluid limitations, worrying about starting dialysis, and concerns about feeling like a burden to their caregivers [119,120]. Depression is a particularly common mental health challenge among people with CKD. A comprehensive review conducted by Palmer and colleagues in 2013 [121] evaluated depressive symptoms by using questionnaires administered by healthcare professionals for individuals at all stages of CKD. They discovered that the rate of depressive symptoms was the highest among those undergoing dialysis, reaching 39.3%. Among individuals with CKD, including those at stages 1–5, and among kidney transplant recipients, about 26.5% and 26.6%, respectively, reported experiencing symptoms of depression. Beyond the emotional distress that comes with these symptoms, it is worth noting that they are also linked to negative health consequences. When individuals with CKD also grapple with depression, it often leads to a lower overall quality of life. They tend to see a faster decline in their kidney function, measured as estimated glomerular filtration rate (eGFR), which accelerates the progression to ESRD. Additionally, they face a greater likelihood of hospitalization and a higher risk of mortality [122,123,124,125]. Information gathered from the CRIC study showed that the presence of depressive symptoms varied depending on the level of kidney function. Specifically, as kidney function, as measured by eGFR, decreased, the chances of experiencing depression became more likely [126].

### 7.3. On Taste Perception

Researchers had indicated the disturbance of taste (5 basic taste qualities) in CKD patients [127,128,129]. There are several factors believed to affect the sense of taste in CKD. These include the impact of kidney-related issues, the use of medications, changes in saliva composition, and variations in dietary habits and nutrition status. Additionally, factors like zinc deficiency can play a role in this as well [130]. Changes in taste are quite common in CKD, and they can influence how much people enjoy their food and what they choose to eat, which, in turn, can impact their nutritional well-being. Research has shown that these taste alterations can happen in CKD regardless of a person’s age or gender. Specifically, there tends to be difficulty in detecting sour, umami, and salty tastes. Manley et al. (2012) [129] and McMahon et al. (2014) [131] had reported that consuming more salt in your diet might raise the amount of sodium in your saliva for CKD patients. This, in turn, could make it harder for them to taste salt, as well as affect their ability to perceive other tastes due to increased solutes in their saliva, including sodium. These factors might have an impact on how someone perceives the overall flavor and how much they enjoy certain foods. For instance, difficulties in detecting umami could affect their interest in protein-rich foods, while trouble with salty taste perception might influence their salt intake. The information about how common taste problems are and what they are like for CKD patients can help guide future efforts to improve their sense of taste. It is important to note that the existing data on taste issues in CKD is somewhat limited. Most of the studies involved small groups of participants, and they mainly focused on individuals in the later stages of CKD.

## 8. Carbohydrate Diet in CKD

Diet plays a crucial role in the management of CKD, and understanding the impact of carbohydrate intake on insulin metabolism and overall health is essential. CKD is associated with significant alterations in insulin metabolism, which can have deleterious effects on an individual’s health. As renal function declines, the clearance of insulin is impaired, leading to higher insulin levels in the bloodstream. This insulin resistance can disrupt glucose homeostasis and exacerbate metabolic complications, including diabetes. Moreover, the altered carbohydrate metabolism in CKD can contribute to the accumulation of AGEs, which are known to be detrimental to various organs and tissues. Therefore, a carefully balanced carbohydrate intake is crucial for CKD patients to manage insulin metabolism, prevent metabolic complications, and promote overall health [132,133].

Individuals with CKD face challenges related to impaired kidney function, including the regulation of certain nutrients. Managing carbohydrates in their diet is crucial for maintaining overall health and mitigating complications associated with CKD [134]. In a carbohydrate-restricted diet for CKD, it is essential to prioritize low-glycemic carbohydrates. These include whole grains, vegetables, and legumes, which are digested more slowly, resulting in gradual increases in blood sugar levels. Unlike high-glycemic carbohydrates, low-glycemic options help avoid rapid spikes in blood sugar, which can be detrimental for individuals with CKD. Controlling portion sizes is another critical aspect of managing carbohydrate intake [135]. This not only regulates blood sugar levels, but also prevents excessive calorie consumption, contributing to weight management—crucial for individuals with CKD to reduce the risk of complications [136]. Complex carbohydrates should be favored over simple sugars. Whole grains, vegetables, and legumes not only provide carbohydrates but also offer fiber and essential nutrients. Fiber is beneficial for digestive health and aids in blood sugar control. Potassium and phosphorus are minerals that individuals with CKD often need to limit. Some carbohydrate-rich foods, such as bananas and potatoes, are also high in potassium. Therefore, monitoring and managing the intake of these minerals is crucial for preventing electrolyte imbalances and supporting kidney health. Individualized diet plans are paramount in CKD management [137]. The severity of CKD varies among individuals, necessitating personalized dietary recommendations. Collaborating with a registered dietitian or healthcare professional ensures a tailored approach, considering specific health conditions and nutritional needs [137]. Fluid intake management is intertwined with carbohydrate consumption [138]. Many carbohydrate-rich foods, such as fruits and vegetables, also contribute to overall fluid intake. Given that fluid restriction is common in CKD, individuals must be mindful of their fluid consumption from both food and beverages [138]. Individualized management gains prominence with CKD progression and among dialysis patients, particularly those with reduced urine output. Fluid requirements’ variability across CKD stages underscores the need for nuanced recommendations. Advanced CKD stages, marked by compromised renal function, demand stringent fluid control to prevent complications. Dialysis patients, especially with limited urine production, face unique challenges, warranting heightened focus on tailored interventions. Beyond CKD, comorbidities like heart failure add complexity to fluid management, necessitating a delicate balance to prevent exacerbating symptoms. This highlights the interdisciplinary nature of CKD care, urging healthcare providers to coordinate interventions for comprehensive and effective patient management. Processed foods, often high in sodium and phosphorus, should be limited. Opting for whole, fresh foods and home-cooked meals enables better control over nutrient intake, supporting kidney health [139]. In conclusion, a carbohydrate-restricted diet in CKD involves selecting low-glycemic, complex carbohydrates, controlling portion sizes, monitoring potassium and phosphorus intake, individualizing dietary plans, managing fluid intake, and minimizing processed foods. Always consult with healthcare professionals for personalized guidance, ensuring the dietary approach aligns with the individual’s unique health status and requirements [137].

## 9. Future Recommendation

In the evolving landscape of nutrition science, the intersection of carbohydrate intake, insulin metabolism, and CKD demands a nuanced and individualized approach to dietary planning. The imperative lies in recognizing the intricate interplay between CKD and insulin dysfunction, understanding the repercussions on overall health, and tailoring carbohydrate consumption to each patient’s distinct needs and metabolic profile. Central to this future direction is the recognition of the adverse effects CKD imposes on insulin release and the broader metabolic landscape. As CKD disrupts the delicate balance of insulin sensitivity, there is a pressing need to shift away from generalized dietary recommendations and embrace a patient-centric paradigm. Monitoring glycemic control and assessing insulin sensitivity become crucial components of this personalized approach, enabling healthcare providers to fine-tune carbohydrate intake based on individual responses. A critical aspect of future recommendations involves a strategic emphasis on the quality of carbohydrates chosen. Opting for complex carbohydrates with a low glycemic index emerges as a key strategy to mitigate postprandial glucose spikes, thereby reducing the risk of insulin resistance. This shift in focus underscores the importance of choosing nutrient-dense, fiber-rich sources over processed sugars, promoting sustained energy release and metabolic stability in CKD patients. Furthermore, dietary patterns should be reimagined to incorporate whole foods, emphasizing the consumption of fresh fruits, vegetables, and whole grains. The inclusion of fiber-rich sources not only aids in glycemic control but also contributes to overall gut health, a factor increasingly recognized for its role in metabolic outcomes. Limiting processed sugars becomes paramount to curbing the accumulation of AGEs, which are implicated in the progression of CKD. Collaborative efforts among healthcare providers, dietitians, and patients are envisioned as essential in translating these nuanced dietary strategies into actionable plans. The complexity of CKD-related insulin dysfunction requires a multidisciplinary approach, fostering a partnership where medical expertise combines with personalized nutritional guidance. This collaborative model ensures that dietary plans are not only effective but also sustainable, accounting for the unique challenges posed by CKD and contributing to improved health outcomes. In conclusion, the future direction of managing carbohydrate intake in the context of insulin metabolism and CKD is inherently personalized and multidisciplinary. By tailoring dietary plans to individual needs, prioritizing complex carbohydrates, and promoting collaborative efforts, the approach aims not only to mitigate the challenges posed by CKD-related insulin dysfunction but also to foster a holistic improvement in metabolic outcomes and overall health.

## Data Availability

Data sharing is not applicable.
